# Neurological and Cognitive Performance After Childhood Encephalitis

**DOI:** 10.3389/fped.2021.646684

**Published:** 2021-04-06

**Authors:** Heidi Pöyhönen, Sirkku Setänen, Nea Isaksson, Mikko Nyman, Anna Nyman, Ville Peltola, Tuire Lähdesmäki

**Affiliations:** ^1^Department of Pediatric Neurology, Turku University Hospital, Turku, Finland; ^2^Departments of Pediatrics and Pediatric Neurology, University of Turku, Turku, Finland; ^3^Department of Women's and Children's Health, Uppsala University, Uppsala, Sweden; ^4^Department of Radiology, Turku University Hospital, Turku, Finland; ^5^Department of Psychology, Turku University Hospital, Turku, Finland; ^6^Department of Social Research, Turku University Hospital, Turku, Finland; ^7^Department of Pediatrics and Adolescent Medicine, Turku University Hospital, Turku, Finland

**Keywords:** central nervous system infection, full-scale intelligence quotient, long-term outcome, minor neurological dysfunction, pediatric encephalitis, the Touwen examination, Wechsler intelligence scale

## Abstract

**Background:** Children with encephalitis have increased risk for long-term neurological sequelae. We investigated minor neurological dysfunction (MND) and cognitive performance as a measurement for long-term outcome of encephalitis in childhood.

**Materials and Methods:** Children with encephalitis (*n* = 98) treated in Turku University Hospital during the years 1995–2016 were retrospectively identified. We included the patients without severe developmental delay before the encephalitis and without recorded neurological disability caused by encephalitis. MND was assessed using the Touwen examination. Age-appropriate Wechsler Intelligence Scale was used to determine the full-scale intelligence quotient (IQ). Residual symptoms in everyday life were evaluated using a questionnaire.

**Results:** Forty-two subjects participated in the study and returned the questionnaire regarding residual symptoms. The median age was 4.3 years at the time of encephalitis, and 11.3 years at the time of the Touwen examination (*n* = 41) and the cognitive assessment (*n* = 38). The Touwen examination indicated MND in 29 of 41 participants (71%; simple MND in 16 and complex MND in 13 patients). The median full-scale IQ was lower in participants with MND compared with participants without MND (98 vs. 110, *p* = 0.02). Participants with IQ < 85 (*n* = 5) had lower median age at acute encephalitis compared to participants with IQ ≥ 85 (*n* = 33) (1.8 vs. 5.3 years, *p* = 0.03). Problems in daily performance were reported in participant with MND (*p* = 0.2) and low full-scale IQ (*p* = 0.008).

**Conclusions:** The prevalence of MND was high and it was related to lower cognitive performance after childhood encephalitis. Younger age at acute encephalitis was a risk factor for lower cognitive performance.

## Introduction

Encephalitis is a severe infective or inflammatory disorder of the brain with many possible etiologies. Children with encephalitis have a risk for long-term neurological sequelae, such as problems in psychomotor speed, memory functions, academic skills, concentration and behavior regulation, as well as focal motor deficits and personality changes (1–6).

Risk factors for poor outcome in earlier studies have varied. Certain viruses, like herpes simplex virus, are known to cause severe neurological sequelae (7). In some studies, poor outcome has been linked to younger age (7–10), while in some others, to older age (11). Abnormal neuroimaging (4, 12), pleocytosis (13) or higher cerebrospinal fluid (CSF) leukocyte level (14) have been reported to be risk factors in some studies, but not in others (9, 10). Additionally, the signs of severe disease, e.g., duration of hospital stay (4, 10), admission to intensive care unit (ICU) (3, 5, 15), or seizures at admission (9, 10, 16) are reported as risk factors for neurological sequelae in many studies.

The poor outcome after childhood encephalitis is well-known, and severe sequelae are usually well-recognized. However, less severe sequelae may remain unrecognized on standard pediatric examination, leaving these patients without sufficient follow-up and support. Less severe symptoms may become evident with age and increasing demands, and cause problems affecting the individual's health, quality of life and independence in everyday life.

The Touwen examination is a standardized neurological examination to detect minor neurological dysfunction (MND) in children from 4 years of age (17). The examination has been developed for clinical practice, but is also used in clinical research (17, 18). There are no studies investigating MND after encephalitis, but MND has been shown to relate to cognitive (19, 20) and behavioral (19) problems in birth cohort studies, which followed up the development of newborn babies to 9 and 12 years of age.

Our aim was to investigate the long-term neurological and cognitive performance after childhood encephalitis in patients without evident disability during initial follow-up. We also investigated the relation of selected risk factors to studied long-term neurological and cognitive outcomes. Additionally, we evaluated subjects' performance in everyday life using self or parent-rated questionnaire. Our hypothesis was that among patients surviving childhood encephalitis without evident disability, the rate of MND would be higher than in general pediatric population. We also hypothesized that MND after pediatric encephalitis would relate to lower cognitive performance and affect child's everyday life functioning.

## Materials and Methods

### Participants

**Figure 1:**
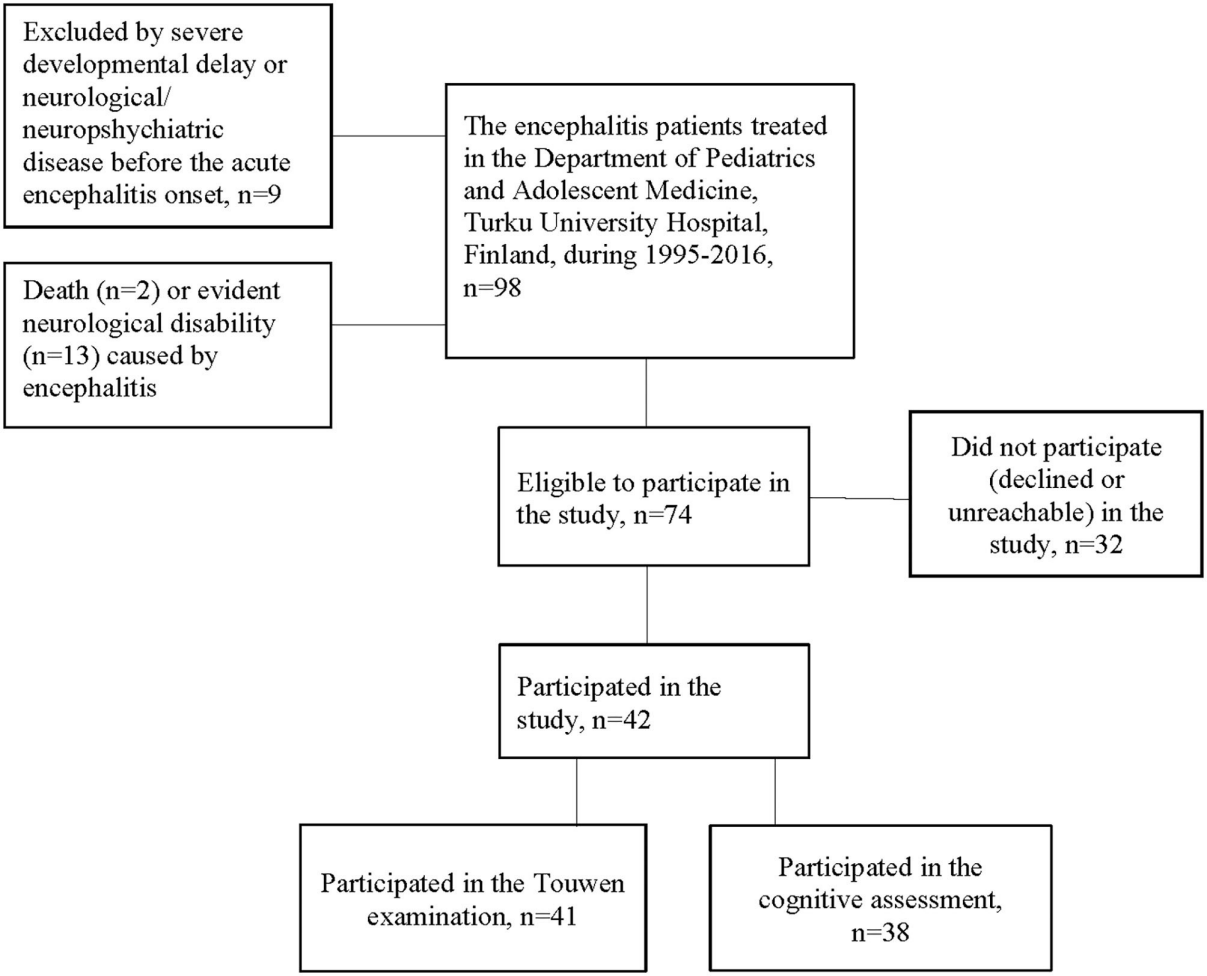
The participant flow-chart.

We identified the patients treated for acute encephalitis (*n* = 98) at age 0–16 years at the Department of Pediatrics and Adolescent Medicine, Turku University Hospital, Finland, during years 1995–2016. Patients were identified from the medical records by the International classification of diseases, 10th Revision (ICD-10) codes, referring to diagnosis of acute central nervous system infection: A80-89, G00-09, and G51-52. Medical records of all patients with one or several of above-listed diagnoses were examined to confirm or exclude the diagnosis. To study MND after encephalitis, only subjects without severe developmental delay (learning or motor disabilities), or any neurological or neuropsychiatric disease before the encephalitis; and without evident neurological disability caused by encephalitis, were eligible to participate in the study. The neurological and cognitive assessments were performed during one or two visits in Turku University Hospital, in 2017. The flow chart is shown in Figure 1.

The diagnosis of encephalitis was defined by acute encephalopathy (depressed or altered level of consciousness or altered behavior lasting ≥ 24 h) occurring with or without findings of acute neurological defect, with previous or concurrent symptoms suggesting an infectious disease (e.g., fever, or respiratory or gastrointestinal symptoms), and either CSF, electroencephalogram (EEG) or neuroimaging findings consistent with encephalitis. CSF findings regarded as consistent with encephalitis were pleocytosis (CSF leukocyte count above 5 × 10^6^/L), elevated protein concentration (>400 mg/L in 2.0–13.9 years old children and 450 mg/L in 14.0–15.9 years old children), or positive polymerase chain reaction (PCR) test for known causative microbial agent of encephalitis. To confirm the radiological findings consistent with encephalitis, acute phase brain magnetic resonance images (MRI) were re-evaluated for any encephalitis related pathology by neuroradiologist (M. N.). Cortical or basal ganglia oedema, diffusion restriction, hemorrhage, hydrocephalus and parenchymal, meningeal or cranial nerve enhancement were considered to be encephalitis related pathology on MRI. General and/or focal disorder with or without epileptiform activity were regarded as EEG findings consistent with encephalitis.

Microbial etiology was investigated by standard laboratory methods in use at the time of patient's disease and findings were divided into two groups: confirmed/probable or uncertain/unknown. Microbial etiology was defined as “confirmed” when there was a positive PCR or IgM antibody result in the CSF for a typical encephalitis-causing pathogen. The etiology was defined as “probable” when there was a positive CSF antigen test for any pathogen; an antibody response in the CSF for an atypical encephalitis pathogen; or a positive IgM antibody result in a single serum sample, a seroconversion, or an increase in IgG antibody levels in paired sera for a typical encephalitis-causing pathogen. Also, in a case with an onset of clinically diagnosed varicella 4 days before development of encephalitis, the etiology was defined as probable. The etiology was defined as “uncertain” when a potentially encephalitis-causing pathogen was detected by PCR or antigen test from nasopharyngeal, pharyngeal, tracheal or fecal specimen, or by high IgG antibodies in a single serum sample.

### Neurological Outcome

The latest version of the Touwen examination was used for the assessment of MND by an experienced physician (H.P). The examination included eight domains: posture and muscle tone, reflexes, involuntary movements (athetoticform movements, choreiform movements, and tremor), coordination and balance, fine manipulation, associated movements, sensory function, and cranial nerve function. The domains were classified as dysfunctional according to the criteria of the manual using computerized scoring. From 4 years of age until the onset of puberty, simple MND was defined as the presence of one or two dysfunctional domains, and complex MND as the presence of more than two dysfunctional domains. The presence of an isolated dysfunctional domain in reflexes did not qualify for the classification of simple MND. After the onset of puberty, simple MND was defined as isolated presence of dysfunctional posture and tone regulation; choreiform dyskinesia; excessive associated movements; mild sensory dysfunction; or mild cranial nerve dysfunction, and complex MND was defined as presence of mild coordination problems or fine manipulative disability, with or without other domains of dysfunction (17).

The examinations were videotaped (K. M.) and classified together with experienced physicians (S. S., T. L.) in order to ensure a consensus regarding the details of the assessments. According to results, the patients were divided into two groups: normal or MND (simple or complex).

### Cognitive Outcome

The cognitive outcome was assessed by an experienced psychologist (N. I.) using Finnish or Swedish translations of age-appropriate Wechsler intelligence scales: Wechsler Preschool and Primary Scale of Intelligence – Third Edition, WPPSI-III (21, 22) (*n* = 10); Wechsler Intelligence Scale for Children – Fourth Edition, WISC-IV (23, 24) (*n* = 17) or Wechsler Adult Intelligence Scale – Fourth Edition, WAIS-IV (25) (*n* = 11). Full-scale IQ (mean ±SD: 100 SD ± 15 in the normative population), which was used in this study as a measure of general intelligence, is a composite score of more specific cognitive domains. Abbreviated versions were used for estimating full-scale IQ in this study for 84% of the patients (32/38). An abbreviated version of the WPPSI-III (21, 22) included two of three subtests (Information and Word Reasoning) from the verbal scale, two of three subtests (Block Design and Picture Concepts) from the performance scale and one (Coding) from the processing speed scale. The abbreviated versions of the WISC-IV (23, 24) and the WAIS-IV (25) included two of three subtests (Similarities and Vocabulary) from the verbal scale, two of three subtests (Block Design and Matrix Reasoning) from the perceptual scale, one of two subtests (Digit Span) from the working memory scale and both two subtests (Coding and Symbol Search) from the processing speed scale. Subtests were selected according to general clinical practice (26) and the scores for abbreviated assessments were assigned according to test manuals (21–25). A cut-off of ≥85 (−1 SD) was considered as average cognitive performance when dichotomous variable of full-scale IQ was used.

### Encephalitis Study Questionnaire

At the study visit, the participants or their parents were asked to fill in a questionnaire consisting of their or their child's medical and developmental history, including comorbidities, medication, neurodevelopment, learning ability, educational background, school performance, sporting activities during follow-up, and any deficits in motor skills and daily performance before and after the encephalitis. Questions regarding daily performance included activities of daily living: dressing, eating, personal hygiene, understanding the concepts of time and value of money as well as independent activity in social environment at age-appropriate level. Difficulties were defined as a subjective experience by the participant or his/her parent. The questionnaire was designed for the purposes of the present study, and is shown in Supplementary Material.

### Statistical Analysis

Following groups were compared: participating and non-participating subjects, participants with normal Touwen examination and MND, and participants with full-scale IQ ≥85 and <85. Differences in continuous characteristics between groups were studied using a Mann-Whitney U-test as the data were not normally distributed. For the categorical characteristics, a chi-square test (*n* ≥ 5) or Fisher's exact test (*n* < 5) were used. Statistical analyses were performed using SPSS version 27.0 (IBM SPSS Statistics, IBM Corporation, NY, USA). A *p*-value of < 0.05, two-tailed, was considered statistically significant.

### Ethics

The study was approved by the Institutional Review Board at the Clinical Research Centre of the Turku University Hospital and the Ethics Committee of the Hospital District of Southwest Finland (71/2016). The participants and/or their parents gave informed consent.

## Results

Of 74 subjects invited to the study, 42 (57%) participated (Figure 1). The 32 subjects who did not participate in the study were older (median 11.0 years; range, 10 months−15.9 years) than the participants (*p* = 0.003). Table 1 shows the clinical characteristics during acute encephalitis in participating and non-participating subjects. The median age of participants at the onset of encephalitis was 4.3 years (min. 3 months, max. 15.7 years). One participant had nephrotic syndrome and immunosuppressive treatment whereas others had no significant somatic diseases prior to encephalitis. Six participants were born prematurely (<37 gestational weeks), two of them very prematurely (<32 gestational weeks). None of prematurely born participants had developmental delay or neurological/neuropsychiatric diseases before the encephalitis onset. One participant had two separate episodes of infection-associated encephalitis, later classified as acute necrotizing encephalopathy due to RANBP2-mutation (27).

**Table 1:** Clinical characteristics during acute encephalitis in subjects participating in the study (*n* = 42), and in subjects not participating in the study (*n* = 32).

	**Subjects participating the study, *n* = 42**	**Subjects not participating, *n* = 32**	***P*-value**
Gender, male, *n* (%)	22 (52.4)	19 (59.4)	0.5
Age at encephalitis onset median (range), years	4.3 (0.3–15.7)	11.0 (0.8–15.9)	0.003
CRP <40 mg/ml, *n* (%)	38 (90.5)	29/30 (96.7)	0.4
Pleocytosis, *n* (%)	32 (76.2)	19/31 (61.3)	0.2
Confirmed or probable microbial etiology, *n* (%)	15 (35.7)	13 (40.6)	0.7
CSF leukocyte count, median, cells E^6^/liter (range)	21 (0–247)	11 (0–635)	0.5
Abnormal EEG, *n* (%)	28/36 (77.8)	24/29 (82.8)	0.6
Abnormal MRI, *n* (%)	21/38 (55.3)	12/26 (46.2)	0.5
Seizures during acute phase, *n* (%)	14 (33.3)	9/31 (29.0)	0.7
Hospital stay, median (range), days	8 (1-75)	6 (1-35)	0.3
Need for ICU, *n* (%)	19 (45.2)	17 (53.1)	0.5
Symptoms at discharge, *n* (%)	26 (61.9)	16 (50.0)	0.3
Duration of acute neurological symptoms > 1 month, *n* (%)	11/41 (26.8)	4/29 (13.8)	0.2
Duration of acute neurological symptoms > 3 months, *n* (%)	7/41 (17.1)	4/30 (13.3)	0.8

*CRP, C-reactive protein; CSF, cerebrospinal fluid; EEG, electroencephalogram; MRI, magnetic resonance imaging; ICU, intensive care unit*.

Brain MRI had been performed in the acute phase (<2 months) of encephalitis for 38 of participants and was normal in 17 (45%) and abnormal in 21 (55%). Only typical oedemic gray and white matter lesions or diffusion restrictive lesions, based on appearance and location, were considered to be related to acute encephalitis. Hemorrhagic lesions and parenchymal enhancement were always associated with other typical lesions. Isolated meningeal enhancement was found in one participant. The association of location, extend or type of these lesions and MND or IQ ratings was not studied due to the small sample size.

The follow-up time from acute encephalitis to study visits was median 6.0 (min, 0.4; max, 22.6), mean 6.7 (SD, 5.4) years. A total of 41 subjects participated in the Touwen examination. The examination was normal in 12 (29%). Simple MND was detected in 16 (39%), and complex MND in 13 (32%) participants. The clinical characteristics during acute encephalitis shown in Table 1 did not differ between these groups.

A total of 38 subjects participated in the cognitive assessment. The median full-scale IQ was 100 (52–106). The full-scale IQ was ≥85 in 33 (87%) and <85 in 5 (13%) of the participants, as shown in Table 2. The full-scale IQ was <70 (52 and 59) in two (5%) participants. Both were born full-time, and suffered encephalitis at early age, one at 5 months and one at 7 months age. The cognitive assessment was performed for one at 7.1 and for the other at 10.9 years of age. The median age at acute encephalitis was significantly lower in participants with full-scale IQ <85 compared to the participants with full-scale IQ ≥ 85 (1.8 and 5.3 years, respectively, *p* = 0.03). The other clinical characteristics during acute encephalitis shown in Table 1 did not differ between these groups. The median full-scale IQ was lower in participants with MND (simple or complex) compared to participants with normal Touwen examination (98 and 110, range 52–112 and 88–126, *p* = 0.02), as shown in Table 3. The results were consistent without two participants with full-scale IQ <70 (52 and 59).

**Table 2:** Demographic and clinical data of subjects participating in the study (*n* = 38) divided into groups by full-scale intelligence quotient (IQ).

	**Full-scale IQ**		***P*-value**
	**≥85, *n* = 33**	** <85, *n* = 5**	
Age at encephalitis, median (range), years	5.3 (0.3–15.7)	1.8 (0.4–3.8)	0.03
Age at study, median (range), years	11.5 (2.3–28.6)	9.8 (7.1–11.0)	0.4
Follow-up time, median (range), years	5.1 (0.4–22.6)	6.7 (4.0–10.3)	0.2
The Touwen examination, minor neurological dysfunction, *n* (%)	21/32 (65.6)	5 (100.0)	0.3
Reported difficulties in motor skills, *n* (%)	3 (9.1)	1 (20.0)	0.4
Reported problems in activities of daily living, *n* (%)	6 (18.2)	3 (60.0)	0.008
Sporting activity, *n* (%)	16/31 (51.6)	4 (80.0)	0.4
**Education**			
At preschool or at primary school, *n* (%)	21/32 (65.6)	5 (100.0)	
Trade school, *n* (%)	4/32 (12.5)	0 (0.0)	
High school, *n* (%)	4/32 (12.5)	0 (0.0)	
University, *n* (%)	3/32 (9.4)	0 (0.0)	
Need for special support at preschool and/or at primary school, *n* (%)	7/32 (21.9)	4 (80)	0.02

IQ, intelligence quotient.

**Table 3:** Demographic and clinical data of subjects participating the study (*n* = 41) divided into groups by the Touwen examination.

	**The touwen examination**		***P*-value**
	**Normal, *n* = 12**	**Minor neurological dysfunction, *n* = 29**	
Age at encephalitis, median (range), years	5.8 (1.8–15.7)	3.8 (0.3–15.4)	0.3
Age at study, median (range), years	13.0 (4.2–28.6)	11.0 (4.4–29.4)	0.8
Follow-up time, median (range), years	4.7 (1.1–22.6)	6.1 (0.4–18.5)	0.8
Full scale IQ, median (range)	110 (88–126)	98 (52–112)	0.02
Full scale IQ <85, *n* (%)	0/11 (0.0)	5/26 (19.2)	0.3
Reported difficulties in motor skills, *n* (%)	1 (8.3)	4 (13.8)	1.0
Reported problems in activities of daily living, *n* (%)	1 (8.3)	9 (31.0)	0.2
Sporting activity, *n* (%)	2/11 (18.2)	20/28 (71.4)	0.04
**Education**			
At preschool or at primary school, *n* (%)	7/11 (63.6)	20 (69.0) 11	
Trade school, *n* (%)	1/11 (9.1)	3 (10.3)	
High school, *n* (%)	1/11 (9.1)	3 (10.3)	
University, *n* (%)	2/11 (18.2)	3 (10.3)	
Need for special support at preschool and/or at primary school, *n* (%)	3/11 (27.3)	9 (31.0)	0.7

*IQ, intelligence quotient*.

The neurological follow-up had ended before the study for 10 of 13 participants with complex MND. Three of them reported problems in daily life. Two of the five patients with IQ <85 performed cognitive assessment for the first time at the study visit. They did not report any problems in daily performance. One of the five patients with IQ <85 had performed cognitive assessment several years before the study visit with normal cognitive performance and was not in the neuropsychological follow-up anymore. This patient reported problems in daily performance.

Of 42 participants who returned the encephalitis study questionnaire, five (12%) reported difficulties in motor skills and ten (24%) in daily performance. Twenty-two participants reported playing sports actively during leisure-time. Twenty-seven (64%) participants attended primary school or preschool at the time of the follow-up. Of 15 participants who had finished the primary school by age of 15 years, 14 (93%) were presently studying, or had completed their studies. Twelve participants reported the need for special support at preschool or at school. The educational background was missing from one of the participants. The results of questionnaire by neurological and cognitive outcome groups are shown in Tables 2, 3.

## Discussion

The prevalence of MND after childhood encephalitis was remarkably high, 71%, in our study population of patients without evident disability. In the general pediatric population, the prevalence of minor and complex MND is reported to be up to 20 and 7% (28, 29). The median full-scale IQ was 12 points lower in participants with MND, compared to participants with normal Touwen examination. Our findings represent increased vulnerability of subjects with MND to the cognitive deficits. We found no statistically significant risk factors at the time of acute encephalitis for later MND in our patient cohort; therefore, it seems that the risk for later MND is difficult to predict without systematic follow up.

In our cohort, the subjects with evident disability were not eligible to participate, which may explain the lack of statistically significant risk factors for MND. This supports the findings of previous studies reporting less severe neurological sequelae after encephalitis (2, 30). We didn't study the relations of most complex neuroimaging findings to poor outcome separately, as did e.g., Bykowski et al. (31), which could as well have affected our results. The only risk factor we found to predict lower IQ was the young age at encephalitis. The small sample size (only five subjects with IQ <85) could have influenced the sparsity of statistically significant risk factors to predict lower cognitive performance in our cohort.

The prevalence of IQ <70 and <85 in the general normative population are 2 and 16% (21–25). After pediatric encephalitis those prevalences have been reported to be higher, 13–22% and 18–31% (4, 6). In our study, the exclusion of patients with learning disabilities before the encephalitis onset, as well as exclusion of patients with recorded evident disability due to encephalitis, have influenced the rates of full-scale IQ <70 and <85. In our cohort, the full-scale IQ <85 was noted in five (13%) subjects, so the prevalence of IQ <85 was not higher than in general population. The full-scale IQ <70 was noted in two (5%) participants. Both suffered encephalitis at very young age, which is a known risk factor for poor outcome (7–10). On the other hand, due to young age at encephalitis onset, it is not possible to find out if the early neurological development of these participants was normal or abnormal without encephalitis.

In our study, MND was related with lower IQ, which is in line with a previous study of 9-year-old children at low risk for developmental disorders (20). In previous study, “neurological soft signs” (defined as deviant performance on a motor or sensory test in the neurological status examination) at school-age associated with not only the risk of cognitive, also psychiatric impairment in adolescence (32). Moreover, it is known that the major problem after pediatric encephalitis is not only the lower IQ, but also problems in specific cognitive functions like in memory and attention regulation (2). Additionally, personality changes, headache, fatigue, and irritability may reportedly affect their performance in everyday life (3). Thus, it is important to evaluate specific cognitive functions and behavioral problems especially in encephalitis patients with difficulties in motor performance more widely.

MRI can be normal in the early phase of encephalitis. Six children in our data were scanned during the first 3 days of the symptoms onset and three of these six during the first or second day of the symptoms onset. These children did not receive follow-up imaging later. Result was considered normal, but follow-up MRI could have revealed encephalitis related abnormal findings. MRI findings in acute encephalitis might be radiologically non-specific but usually typical for the disease combined with the clinical picture.

Our diagnostic criteria for encephalitis were strict; only the patients with ≥24 h of encephalopathy with CSF, MRI or EEG abnormality related to encephalitis were included. We wanted to exclude the patients with mild encephalopathy caused by dehydration or hypoglycemia during febrile illness, therefore we required objective findings of central nervous system infection or inflammation for encephalitis diagnosis. In comparison, in other studies the criteria have been less strict (3, 8, 10, 12, 16). This approach is inevitably affecting the sample size as well as the outcome in our study. Clinical diagnosis of encephalitis is often demanding, and some diagnoses prove wrong after follow-up. On the other hand, our strict inclusion criteria might have caused exclusion of less severe encephalitis cases. This might worsen the overall outcome of our patient cohort at group level.

A strength of this study was the use of standardized methods to assess the neurological outcome. To our knowledge, our study is the first to assess MND after pediatric encephalitis. We used the complete protocol of the Touwen examination latest version to obtain detailed and reliable information. The examination was performed and scored by an experienced physician and assessed from video recording by two other experienced physicians to ensure the reliability of the ratings. The reliability of the Touwen examination in the assessment of MND has been shown excellent or good (18). The cognitive assessments were performed by an experienced psychologist, working in the hospital department of pediatric neurology. The subjects not participating in the study did not differ significantly from the study population, except by older age at the onset of encephalitis.

The study has some limitations. The data concerning acute illness was collected retrospectively from the medical records. The questionnaire used was designed for the purposes of the present study without validation. However, there is no standardized questionnaire developed for the follow up after pediatric encephalitis. Time between the encephalitis and our outcome assessment was variable, and for some participants, more than 20 years. Because the participants were at different stages of development, the deficits may affect their everyday life at different levels. At an older age less severe challenges can manifest more clearly. On the other hand, with age a person may develop compensatory mechanisms to manage minor deficits. Two thirds of the non-participating subjects had reached adulthood at the time of the catchment. Younger age has been shown to be a risk factor for poor outcome (7–10), meaning that the higher proportion of younger children may also have influenced by worsening the outcome in our study. In this study, we did not investigate the overall outcome of encephalitis. The focus of this study was on a cohort of patients without recorded evident disability after encephalitis.

## Conclusions

The prevalence of MND was high, and it was related to lower full-scale IQ in children with a history of acute encephalitis. Encephalitis at a younger age was related to lower full-scale IQ. Our results highlight the importance of long-term follow-up of neurological and cognitive development after childhood encephalitis. As the risk factors for poor outcome are not well recognizable at the acute phase of disease, we recommend multidisciplinary follow up for all children after encephalitis. The youngest children have to be followed up with special attention. The Touwen examination can be useful in detecting non-evident neurological deficits and need for cognitive assessment after childhood encephalitis.

## Data Availability Statement

The raw data supporting the conclusions of this article will be made available by the authors, without undue reservation.

## Ethics Statement

The studies involving human participants were reviewed and approved by The Ethics Committee of the Hospital District of Southwest Finland (71/2016). Written informed consent to participate in this study was provided by the participants' legal guardian/next of kin.

## Author Contributions

HP carried out the data collection, and the neurological examinations of patients, participated in the design of the study, statistical calculations, analyses and interpretation of the data, and drafted and reviewed the manuscript for its intellectual content. SS carried out the classification of neurological outcome by videotapes, and the statistical analyses and interpretation of the data, participated in the design of the study, and drafting and revision of the manuscript. NI carried out the cognitive assessments of patients, participated in the design of the study and data collection, and drafting and revision of the manuscript. MN carried out the re-evaluation of brain MRI and participated in the design of the study, and drafting and revision of the manuscript. AN participated in the design of the study, the statistical considerations, and drafting and revision of the manuscript. VP supervised design of the study and statistical considerations, and critically reviewed the manuscript for its intellectual content. TL carried out the classification of neurological outcome by videotapes, conceptualized and designed the study, supervised the data acquisition and statistical considerations, and critically reviewed and revised the manuscript for its intellectual content. All authors contributed to the article and approved the submitted version.

## Conflict of Interest

The authors declare that the research was conducted in the absence of any commercial or financial relationships that could be construed as a potential conflict of interest.
