# Supplementary material for: Neurological and Cognitive Performance After Childhood Encephalitis
Source: Front Pediatr. 2021 Apr 6;9:646684. doi: 10.3389/fped.2021.646684 (PMC8055844; doi:10.3389/fped.2021.646684)
Supplement: Supplementary file 1 [file Data_Sheet_1.PDF]

QUESTIONNAIRE (Childhood Encephalitis Research Project)

(a translation from Finnish questionnaire)

General information of the participant:

Name:____

Date of birth:____

Age now: ____________ Age at the onset of central nervous system infection: _________________

Birth weight/ height / head circumference:____

Hobbies: ____

A family history of CNS diseases (e.g. developmental disability, epilepsy, central nervous system infections):

____

____

Neurological development in childhood:

Motor skills:

Age when started walking with/without support:__________________

Need for physiotherapy (circumscribe): yes no

Speech:

Age when started saying single words: _______________________

Age when started saying sentences (2-3 words one after another):___________________

Need for speech therapy (circumscribe): yes no

Learning skills:

Any behavioral or learning difficulties at preschool (please describe):

____

____

____

Any behavioral or learning difficulties at school (e.g. social problems, attention problems, or need for support, an assistant or individualized curriculum etc.) (please describe):

____

____

____

Education

Present place of study (circumscribe and complete):

Primary school   Class grade: _______________________________

High school

Trade school   Field of study: _______________________________

University   Main subject: _______________________________

Present learning difficulties (please describe):

____

____

____

Earlier education, if any:

____

Working life

Current status (circumscribe):

Not yet in working life (still at school or studying)

Working occasionally Employer/working place:____________________________

Permanent job Employer/working place:____

Unemployed

Disabled to work

General health

Diseases and medications **before** the central nervous system infection: __________________

____

____

Diseases and medications **after** the central nervous system infection: ___________________

____

____

Present diseases and medication: ____

____

____

Present difficulties in **motor skills** at home/school/hobbies yes no

Present difficulties in **activities of daily living** (circumscribe)

Needs help with washing, eating, dressing etc.	yes	no
Needs help moving outside the home	yes	no
Difficulties understanding the time or the value of money	yes	no
Needs continuous personal assistance (can´t be left alone)	yes	no

Other difficulties (please describe):

____

____

____

Do you think the central nervous system infection changed you (the participant)/your child permanently (e.g. behavior, cognition, motor skills etc.)? Please describe:

____

____

____

____

Previous cognitive assessments:

When:____

Where: ____

The name of the psychologist: ____

Who filled in this form (circumscribe)

Parent

Participant

Other; who: ____

Sign: ____

Date: ____
